# Bioinformatic prediction of proteins relevant to functions of the bacterial OLE ribonucleoprotein complex

**DOI:** 10.1128/msphere.00159-24

**Published:** 2024-05-21

**Authors:** Chrishan M. Fernando, Ronald R. Breaker

**Affiliations:** 1 Department of Molecular Biophysics and Biochemistry, Yale University, New Haven, Connecticut, USA; 2 Department of Molecular, Cellular and Developmental Biology, Yale University, New Haven, Connecticut, USA; 3 Howard Hughes Medical Institute, Yale University, New Haven, Connecticut, USA; University of Wisconsin-Madison, Madison, Wisconsin, USA

**Keywords:** bTOR, comparative genomics, MpfA, noncoding RNA, phylogenetics, RNA World

## Abstract

OLE (ornate, large, extremophilic) RNAs are members of a noncoding RNA class present in many Gram-positive, extremophilic bacteria. The large size, complex structure, and extensive sequence conservation of OLE RNAs are characteristics consistent with the hypothesis that they likely function as ribozymes. The OLE RNA representative from *Halalkalibacterium halodurans* is known to localize to the phospholipid membrane and requires at least three essential protein partners: OapA, OapB, and OapC. However, the precise biochemical functions of this unusual ribonucleoprotein (RNP) complex remain unknown. Genetic disruption of OLE RNA or its partners revealed that the complex is beneficial under diverse stress conditions. To search for additional links between OLE RNA and other cellular components, we used phylogenetic profiling to identify proteins that are either correlated or anticorrelated with the presence of OLE RNA in various bacterial species. This analysis revealed strong correlations between the essential protein-binding partners of OLE RNA and organisms that carry the *ole* gene. Similarly, proteins involved in sporulation are correlated, suggesting a potential role for the OLE RNP complex in spore formation. Intriguingly, the Mg^2+^ transporter MpfA is strongly anticorrelated with OLE RNA. Evidence indicates that MpfA is structurally related to OapA and therefore MpfA may serve as a functional replacement for some contributions otherwise performed by the OLE RNP complex in species that lack this device. Indeed, OLE RNAs might represent an ancient RNA class that enabled primitive organisms to sense and respond to major cellular stresses.

**IMPORTANCE:**

OLE (ornate, large, extremophilic) RNAs were first reported nearly 20 years ago, and they represent one of the largest and most intricately folded noncoding RNA classes whose biochemical function remains to be established. Other RNAs with similar size, structural complexity, and extent of sequence conservation have proven to catalyze chemical transformations. Therefore, we speculate that OLE RNAs likewise operate as ribozymes and that they might catalyze a fundamental reaction that has persisted since the RNA World era—a time before the emergence of proteins in evolution. To seek additional clues regarding the function of OLE RNA, we undertook a computational effort to identify potential protein components of the OLE ribonucleoprotein (RNP) complex or other proteins that have functional links to this device. This analysis revealed known protein partners and several additional proteins that might be physically or functionally linked to the OLE RNP complex. Finally, we identified a Mg^2+^ transporter protein, MpfA, that strongly anticorrelates with the OLE RNP complex. This latter result suggests that MpfA might perform at least some functions that are like those carried out by the OLE RNP complex.

## INTRODUCTION

Large noncoding RNAs (ncRNAs) are rare in bacteria (1), but those whose functions are known perform major tasks such as peptide bond synthesis (ribosomal RNAs) (2), RNA phosphoester hydrolysis (RNase P ribozyme) (3), or RNA phosphoester transfer (self-splicing ribozymes) (4–6), among other diverse functions (1, 7). Given the fundamental biochemical roles some of these large ribozymes perform, it has been proposed that they represent modern versions of ancient RNAs that were important during the RNA World (8, 9)—long before protein enzymes and receptors emerged in evolution (10, 11). Additional large and highly structured ncRNA classes have been identified in bacteria (12–14), but their precise biological and biochemical functions remain to be established. These mysterious ncRNA classes possibly provide opportunities to reveal more about the ancient functions of RNA while also establishing how modern cells still rely on molecular relics from the RNA World.

We have been working to establish the functions of OLE RNA (14–23), which was named for its ornate structure, large size, and its presence in extremophilic bacterial species (14). OLE RNAs are abundantly produced by many Gram-positive, spore-forming bacteria that are often defined as obligate or facultative anaerobes (14, 22). These ~600-nt RNAs have unusual characteristics, including a strikingly well-conserved architecture (14, 17), participation in forming a large ribonucleoprotein (RNP) complex (15, 17, 19, 20, 22), and localization at the membrane of host cells (15). Given that the most highly conserved ncRNA classes whose representatives approach or surpass the size and complexity of OLE RNAs are ribozymes, we have proposed that the most likely biochemical purpose of OLE RNAs is to catalyze a chemical reaction (1, 14).

Several approaches have been used to seek clues regarding the biological and biochemical functions of OLE RNA. Frequent genomic location of the *ole* gene immediately adjacent to a gene for a predicted membrane protein of unknown function provided the initial insight needed to eventually establish that OLE RNA forms a 1:2 complex with OLE-associated protein A (OapA) (15). Additional advances were made by exploiting an OLE-containing organism *Halalkalibacterium halodurans* (formerly called *Bacillus halodurans*) for OLE RNA genetic manipulations (24). For example, genetic knockout strains lacking OLE RNA or OapA have revealed the importance of a functional OLE RNP complex for normal growth when cells are exposed to short-chain alcohols (e.g., 5% ethanol) (16), cold (~20°C) (16), high Mg^2+^ (>2 mM) (18), or non-preferred carbon/energy sources (e.g., glutamate) (23), among other diverse phenotypes (22). Similarly, the exploitation of an unusual OapA mutant protein called “OapA PM1” in a genetic suppressor selection revealed the existence of a second protein partner called OapB (17). RNA pull-down analyses revealed the existence of a third protein partner, OapC (21), and numerous other candidate components of the OLE RNP complex. Genetic suppressor selections (17, 18, 23) are revealing numerous other proteins that are candidate partners or that are at least functionally connected to pathways relevant to the operation of this device.

Various clues have been evaluated to derive a general theory regarding the function of OLE RNA. We have speculated (22) that the OLE RNP complex participates in the biological responses to diverse cellular stresses. Specifically, the complex is proposed to be the functional equivalent of eukaryotic mTOR complexes (25, 26) that are central to the regulation of cellular responses to some stresses also relevant to OLE RNP complex function. If these particles serve similar purposes between these two domains of life, then by analogy the OLE RNP complex might be appropriately named the “bTOR” complex (22), although no direct evolutionary homology between the components is implied by this term.

The diversity of processes associated with the OLE RNP complex continues to expand as additional links are made between assorted cellular components and this device. It is anticipated that such connections could be used to further evaluate the bTOR theory while also supplying important clues regarding the possible biochemical functions of OLE RNA. In the current study, we used a comparative genomics approach to search for additional proteins that might be relevant to the OLE RNP complex and its functions. Bacillota species were first sorted based on whether they carry the *ole* gene. A phylogenetic profiling strategy (27) was then used to identify protein-coding genes that are enriched in organisms with *ole* or depleted in organisms lacking this ncRNA gene.

These analyses reveal that the presence of OapA, OapB, and OapC is strongly favored in organisms with *ole*. Similarly, genes relevant to the process of sporulation, among other notable processes, are also correlated with the presence of *ole*. Conversely, the Mg^2+^ transporter MpfA (28–31) is anticorrelated with *ole* within Bacillota species. These results reveal that additional proteins likely serve as physical or functional partners of the OLE RNP complex. Furthermore, the anticorrelation of OapA and MpfA, which are similar in structure, suggests that these proteins might perform some of the same functions. Finally, the results demonstrate that phylogenetic profiling can yield novel insights regarding the functions of ncRNAs.

## RESULTS AND DISCUSSION

### Full-length and variant OLE RNAs are found in distinct clades across Bacillota

The large quantity of bacterial genomic sequence data has allowed researchers to establish detailed phylogenetic trees for much of the known bacterial domain of life (32, 33). These trees have facilitated studies of the evolution of traits across diverse bacterial species. A useful set of data for such efforts is the Genome Taxonomy Database (GTDB) (34), which also has been used to determine functional connections between protein-coding genes (35). Similarly, we use GTDB to determine functional connections between OLE RNA and protein-coding genes.

**Fig 1:**
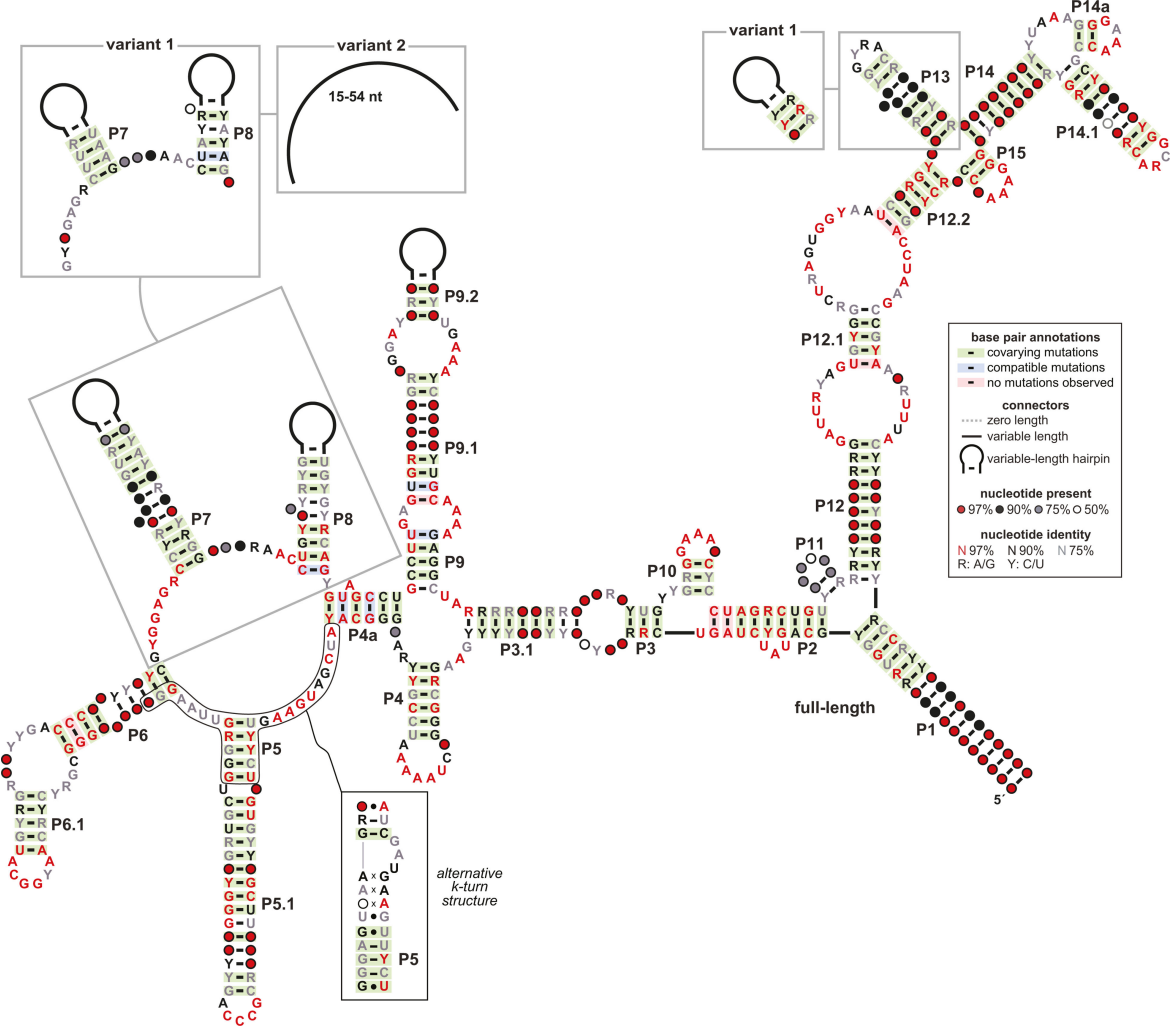
Consensus sequence and secondary structure models for OLE RNAs. The main diagram represents the consensus model for full-length OLE RNAs, which represents the predominant form of the ncRNAs from species in GTDB R08-RS214. Thus, the consensus diagram has some differences with the model for OLE RNA published previously (21). Boxed regions identify the major changes present in the consensus models for variant 1 (TANB77 clade) and variant 2 (RF39 clade) as labeled. The secondary structure model includes an alternative k-turn structure that is formed when OapC is bound to the RNA.

To update the phylogenetic distribution of OLE RNA, we used the RNA homology search algorithm Infernal (36) to computationally identify representatives from bacterial species present in GTDB R08-RS214. This search began with a consensus sequence and secondary structure model derived from an OLE RNA sequence alignment published previously (21). This homology search yielded three general clusters of OLE RNA representatives (Fig. 1) based on how closely the hit matches the previously published model (Fig. S1). We named these three types of OLE RNAs “full-length,” “variant 1,” and “variant 2.”

**Fig 2:**
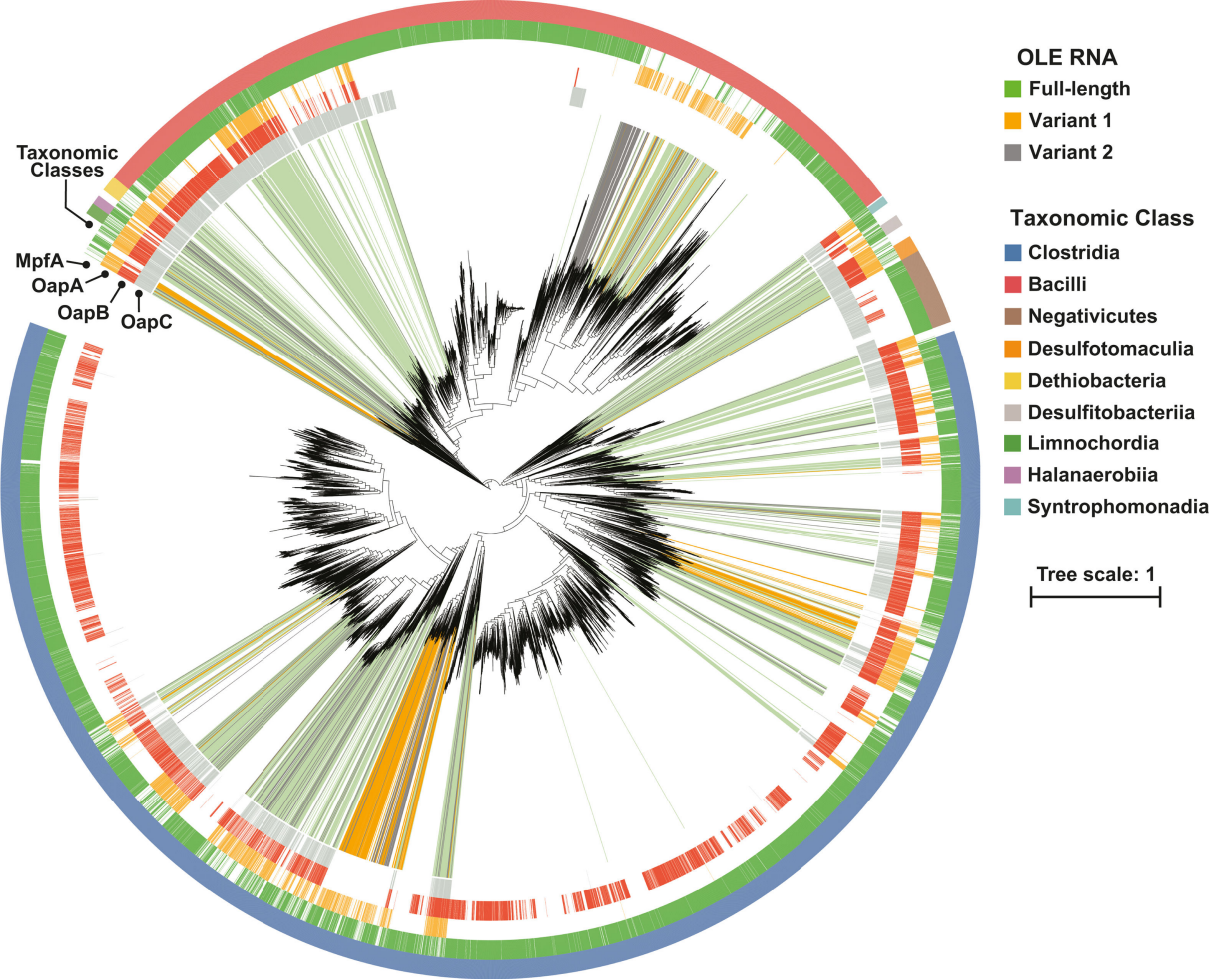
Phylogenetic species tree of Bacillota with correlations and anticorrelations between OLE RNA and four proteins is indicated. The tree was established based on GTDB R08-RS214. Scale units are amino acid substitutions per site. Open regions in the Taxonomic Class bar are classes that are not listed in the figure.

Nearly all OLE RNA representatives are present in species from the superphylum Bacillota (Fig. 2), which GTDB divides into 10 subphyla. Specifically, 2,822 hits reside in Bacillota, whereas only 24 representatives were uncovered in other, more distant phyla. The *ole* genes of the latter hits are typically flanked by DNA sequences with high similarity to genomic regions from organisms in Bacillota, suggesting that these hits might be due to issues such as horizontal gene transfer or errors in genome sequence assembly. Regardless, we chose to exclude non-Bacillota species hits from subsequent analyses.

Most of the OLE RNA representatives are clustered into distinct clades that are located throughout the superphylum (Fig. 2). This distribution suggests that OLE RNAs likely emerged early in evolution, that they serve one or more important functions in certain lineages, and that other lineages have either replaced or otherwise no longer require the OLE RNP complex. Most major taxonomic classes in Bacillota have at least a few species that carry OLE RNA. The only exception is Negativicutes class, which is notable because it represents one of only a few clades in Bacillota that are negative for Gram stain (37). Intriguingly, OapA is a membrane-bound protein, and the OLE RNP complex is membrane associated. This hints at the possibility that the OLE RNP complex is somehow relevant to cell wall biology in some Gram-positive species.

Upon aligning the sequences of the OLE RNA representatives, we observed that both variant classes have many of the same highly conserved nucleotides as full-length OLE RNAs (Fig. 1; Fig. S2). However, OLE RNAs in many clades (but most commonly among TANB77 organisms) have shorter P7, P8, and P9 stems (variant 1) relative to full-length OLE RNAs. Additionally, variant 1 RNAs are often missing nucleotides previously (17, 19, 20) determined to serve as the OapB-binding site at the end of the P13 stem. Variant 2 RNAs, which are also widely distributed but commonly found in RF39 organisms, entirely lack P7 and P8.

Both TANB77 and RF39 clades include uncultured gut microbes that are only known from metagenome assemblies. They are likely obligate symbionts and have highly reduced genomes that are missing genes for many highly conserved metabolic processes (38, 39). These organisms thus might have been subjected to strong evolutionary pressure to reduce the size of their OLE RNAs. Likewise, these organisms are also largely missing OapB and OapC, and perhaps their OLE RNP complexes have fewer essential protein partners. The diminished or missing RNA regions in these species could reflect the loss of one or more features relevant to the function of full-length OLE RNAs. Therefore, we also chose to exclude species with variant OLE RNAs from further analysis in this study.

### OLE RNAs phylogenetically correlate with their known protein-binding partners

**Fig 3:**
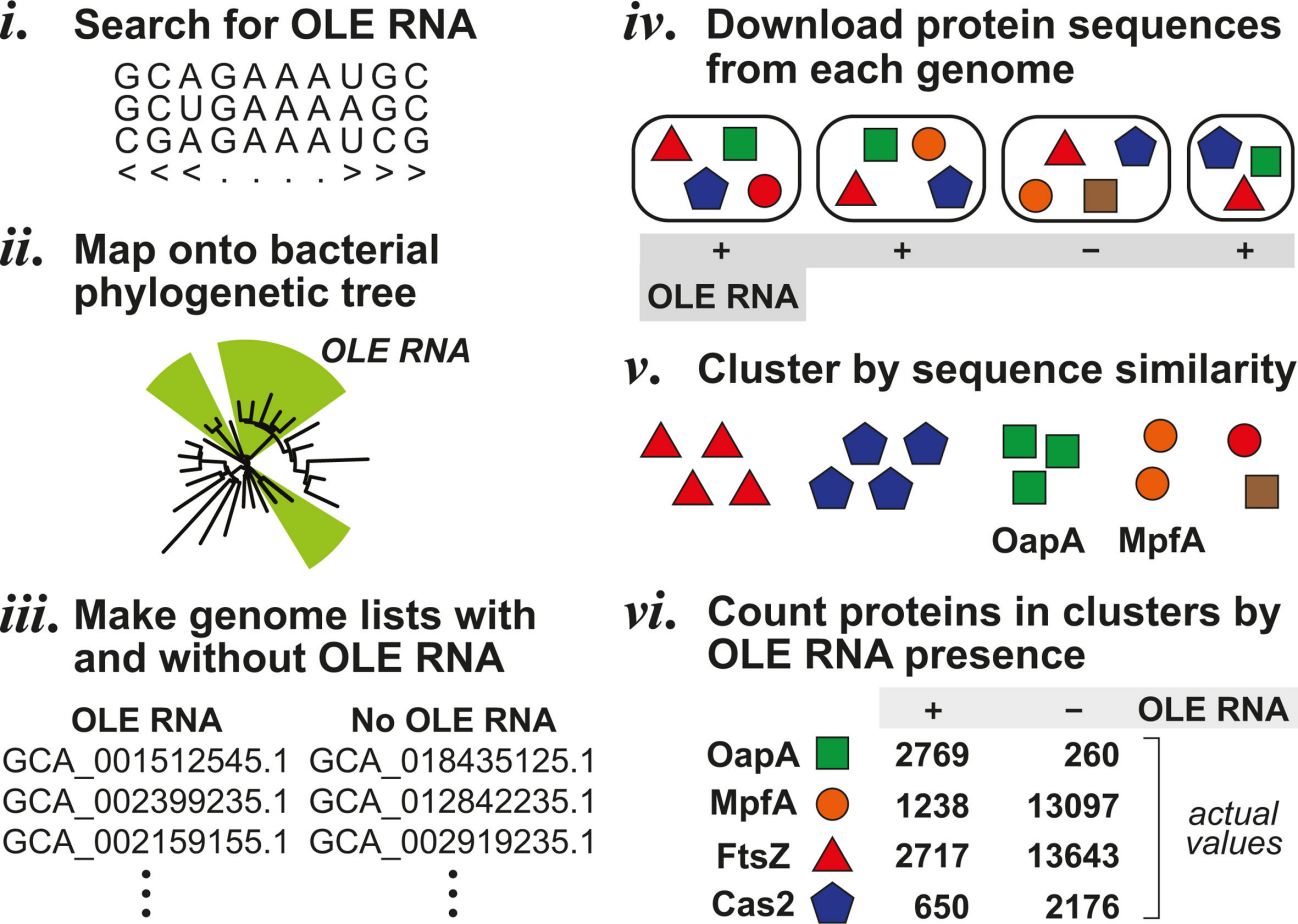
Schematic depiction of the phylogenetic profiling method used to identify proteins whose presence is correlated or anticorrelated with OLE RNA. (i) Infernal is used to search for OLE RNA examples in a database of bacterial genome assemblies specified by GTDB R08-RS214. (ii) Genome assemblies with an OLE RNA gene are mapped onto a bacterial phylogenetic tree of life derived from GTDB. (iii) Genome assemblies are sorted into two groups: those that carry an OLE RNA gene and those that do not. (iv) Protein sequences are downloaded for each genome assembly using the NCBI FTP, or Prodigal is used to predict protein sequences if they are unavailable. (v) Protein sequences from a preselected genome assembly are used as queries for DIAMOND searches with an *e*-value cutoff of 1 × 10^−10^ against the protein sequence database. The resulting groups comprise families of predicted protein homologs. (vi) The approximate number of genome assemblies containing a member of each protein family is determined, and further refinements of the resulting numbers are made as needed. See Materials and Methods and supplemental material for additional details on the computational analyses.

A comparative genomics technique called phylogenetic profiling has been shown to provide valuable information regarding proteins whose functions are uncertain (27, 40). Although phylogenetic profiling has previously been used to identify ncRNA motifs (41), this approach has not been systematically used to associate ncRNAs with protein factors. Regardless, we adapted the phylogenetic profiling strategy to uncover proteins that are either correlated or anticorrelated with OLE RNA. This approach (Fig. 3) builds on our phylogenetic analysis of species that carry full-length OLE RNAs as described above.

After expanding the number of OLE RNA representatives (Fig. 3, step i) and mapping these hits onto the bacterial phylogenetic tree (step ii), we then created two sets of genome assemblies (step iii): those that carry the *ole* gene for full-length OLE RNAs and those that do not carry an *ole* gene for any OLE RNA type. We then used the NCBI FTP (42) to download all annotated proteins encoded by these species (step iv). For genome assemblies that do not include FASTA files with protein sequences, these sequences were predicted using Prodigal (43). Proteins were then clustered by sequence similarity using DIAMOND (44) (step v). Finally, we chose *H. halodurans* to provide a “reference genome” to represent a species with OLE RNA and *Bacillus miscanthi* to provide a reference genome from a species that lacks OLE RNA. These choices were made because *H. halodurans* has served as our model species for OLE RNA genetics and biochemistry studies (14–24), and *B. miscanthi* is its closest relative that lacks OLE RNA. Proteins from these organisms were counted in all genome assemblies from the two lists based on correspondence to the protein clusters (step vi).

The final protein counts were then used to determine whether the presence of each protein is correlated or anticorrelated with the presence of OLE RNA. Two factors prevent the direct comparison of protein counts when assessing correlations with OLE RNA. First, the number of genome assemblies carrying OLE RNA (2,822 genomes) is less than the number of assemblies where OLE RNAs were not readily identified (14,433 genomes). Second, some proteins have more than one representative in some genome assemblies. Therefore, we cannot simply compare the protein counts between the two types of genome assemblies.

**Fig 4:**
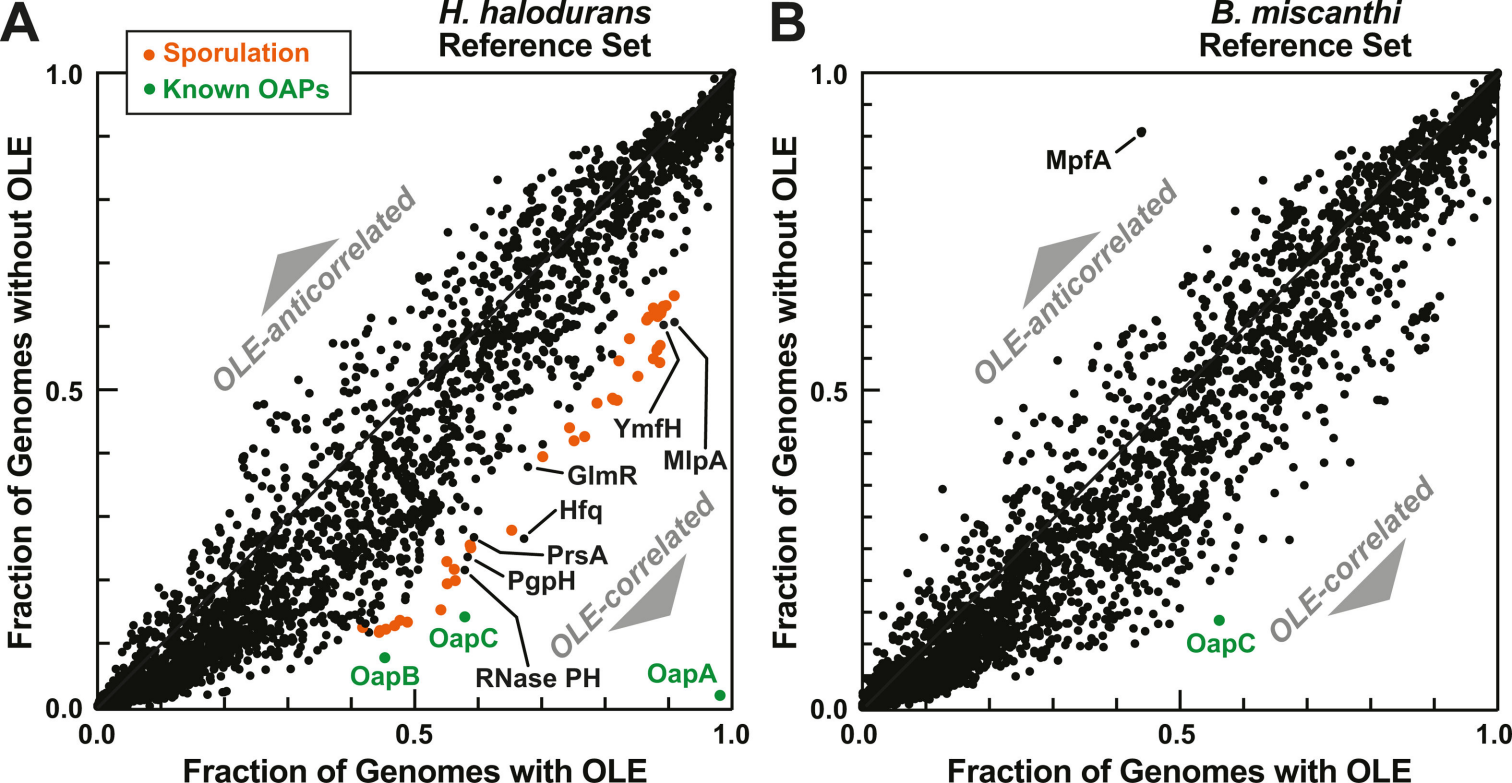
Plots of the distributions of proteins among organisms that carry OLE RNA versus those that lack the ncRNA. (**A**) Distribution of proteins present in *H. halodurans*, which carries an OLE RNA. Notable proteins that positively correlate with OLE RNA are labeled. (**B**) Distribution of proteins present in *B. miscanthi*, which lacks an OLE RNA. MpfA most strongly anticorrelates with OLE RNA.

To address this complexity, further analysis was necessary to identify biases in protein distribution between OLE-containing species and species lacking the ncRNA. Each protein was plotted based on its fractional presence in these two groups (Fig. 4). As expected, the vast majority of proteins present in *H. halodurans* (carries OLE RNA) are not notably biased toward either group, whereas the known OLE-associated proteins (OapA, OapB, and OapC) stand out because a greater fraction of OLE-containing species have them compared to species that lack OLE RNA (Fig. 4A). Furthermore, we conducted a separate analysis of the proteins based on the computation of mutual information (see Materials and Methods for details) and again observed that OapA was the top-ranked protein correlating with OLE RNA (Fig. S2).

OapA was also the most strongly correlated protein when the analyses were modified to avoid the use of a reference genome (reference agnostic) (Fig. S3). This approach was used in an attempt to identify additional proteins of interest that are not present in either of the chosen reference genomes (*H. halodurans* and *B. miscanthi*). The reference agnostic approach was conducted by computationally clustering the full protein database and then performing the remainder of the analysis using representatives from each protein cluster. However, we did not identify additional correlating proteins that were not already identified from the analyses using reference genomes, but some proteins do not appear in both analyses for reasons described in supplemental material.

### OLE RNAs phylogenetically correlate with additional proteins

Numerous other proteins also exhibit a bias in favor of organisms that carry OLE RNA. Over three dozen proteins functionally relevant to sporulation are among the proteins most highly correlated with the presence of OLE RNA (Fig. 4A). It is not certain that all sporulation proteins have been denoted here because some organisms use different sets of factors for sporulation (45, 46). Regardless, the abundance of sporulation factors found to at least modestly correlate with OLE RNA is consistent with the fact that two genes linked to sporulation are among the top 16 genes located near the *ole* gene (22). Because sporulation is a major stress response program (47) and because disabling mutations to components of the OLE RNP complex have revealed links to major stress responses (16, 18, 22, 23), it is also reasonable to speculate that OLE RNAs in many species might be involved in the process of sporulation. Indeed, preliminary results indicate that the disruption of essential OLE RNP components in *H. halodurans* leads to modest sporulation defects (R. R. Breaker et al., unpublished data).

Several other proteins that have not previously been implicated in OLE RNA biology also exhibit a positive correlation (Fig. 4A). Among these is a notable group that includes PrsA, MlpA, and YmfH. The PrsA protein is involved in the folding of secreted proteins (48) and has been shown to interact with the putative metal ion transporter MeeY (formerly YkoY) (31). By genetic suppressor selection, we previously determined that MeeY overexpression can overcome growth deficiency phenotypes in *H. halodurans* strains with an impaired OLE RNP complex (23). In that same study, we also determined that cells with disrupted OLE RNP complexes exhibit a strong protein secretion deficiency. Furthermore, the MlpA protein is known to be involved in protease secretion (49, 50), and YmfH likely has a biochemical function that is similar to that of MlpA (51). Thus, protein secretion is both affected in OLE knockout cells (23) and is a recurring theme among the list of correlated protein factors. As a result, the PrsA, MlpA, and YmfH proteins might be functionally or perhaps even physically associated with the OLE RNP complex.

The remaining noteworthy proteins on the list include GlmR, Hfq, RNase PH, and PgpH-. The GlmR protein is involved in partitioning carbon flow between central carbon metabolism and cell wall biosynthesis (52). Intriguingly, disruption of the OLE RNP complex causes strong growth disruption when *H. halodurans* cells are cultured on non-preferred carbon/energy sources (e.g., glutamate rather than glucose) (23). Cell wall remodeling is required during cell growth and division, and its integrity is important for stress resistance (53). Perhaps OLE RNA might exploit GlmR to modulate carbon utilization and cell growth/division as a mechanism to respond to diverse stresses (22).

Hfq is an RNA-binding protein known in *Escherichia coli* to serve as a folding chaperone for the interaction of RNA partners, particularly involving sRNA-mRNA interactions for the purpose of gene regulation (54–56). The function(s) of Hfq in Bacillota are less well established (57, 58). However, like OLE RNA, Hfq is known to be relevant to stress responses in many species (56, 58), and perhaps the two collaborate in some species to mitigate diverse stresses. RNase PH is another RNA-binding protein that functions as an RNase to process precursor tRNAs (59). OLE RNA, which is known to be processed at its 3′ end in *H. halodurans* (14), has been shown (60) to be susceptible to strand scission by RNase P. Therefore, it seems reasonable to speculate that RNase PH might process precursor OLE RNA transcripts in some species. However, the genes for Hfq and RNase PH are far more widespread than OLE RNA, and their emergence as hits in phylogenetic profiling might be incidental rather than an indication of a functional connection to OLE RNA.

The last protein correlated with OLE RNA discussed herein is PgpH, which is a cyclic-di-AMP phosphodiesterase (61, 62). The bacterial signaling molecule c-di-AMP regulates many cellular processes (62, 63), including several stress responses also associated with the function of OLE RNA (16, 18, 22, 23). It has also recently been found that c-di-AMP can play a role in Mg^2+^ homeostasis (64), for which OLE RNA also plays a role (18). Given other intriguing connections between c-di-AMP and OLE RNA, we have previously proposed that OLE RNA might function as a c-di-AMP synthase ribozyme to perform some of its stress-response functions (22). However, this proposed function and the comments regarding the proteins correlated with OLE RNA are highly speculative at this time.

### The MpfA protein anticorrelates with OLE RNA

Foremost among the proteins that anticorrelate with OLE RNA is MpfA. This protein did not emerge as a signal when *H. halodurans* was used as the reference genome (Fig. 4A) because the *mpfA* gene is absent in this species. In contrast, MpfA exhibits strong anticorrelation when *B. miscanthi* is used as the reference (Fig. 4B). MpfA and orthologs like YhdP have been implicated in Mg^2+^ export (28–30, 65, 66). We previously reported that genetic disruption of the OLE RNP complex causes severe sensitivity to even modestly elevated concentrations (>2 mM) of Mg^2+^ in culture media (18), which is similar to that observed when *mpfA* is deleted (28). This fact, coupled with the observation that MpfA has a CNNM (DUF21) domain that is weakly homologous to OapA, led us to suggest a potential link between MpfA and OapA (18). Because OLE RNA and OapA strongly correlate, and MpfA anticorrelates with OLE RNA, then OapA and MpfA also must anticorrelate. This finding is consistent with the hypothesis that OapA and MpfA are likely to have at least partial functional overlap (18, 22).

To more rigorously examine the distribution patterns of MpfA and the OLE-associated proteins OapA, OapB, and OapC, we created custom hidden Markov models (HMMs) (67) to precisely identify these proteins in various species. In addition, we expanded our analysis to include several MpfA orthologs, such as *Staphylococcus aureus* MpfA (28, 29), *Bacillus subtilis* MpfA (30), *Thermus parvatiensis* CorC (65), and *Methanoculleus thermophilus* CorB (66). These proteins all have similar properties, especially with respect to Mg^2+^ transport. Furthermore, they all have a CNNM domain followed by tandem CBS domains and an HlyC/CorC domain. We reasoned that all proteins with the same domain architecture likely comprise a protein family with the same or similar function. Thus, an HMM was constructed for this protein family, which we refer to simply as “MpfA.”

The HMMs were used to conduct a more sensitive and thorough search of homologs of these proteins than was performed using DIAMOND (44) during the initial phylogenetic profiling analysis. Genome assemblies containing homologs of these proteins were then plotted on the bacterial species tree of OLE-containing organisms (Fig. 2). As expected, these data reveal that OLE RNA and OapA are almost completely mutually inclusive, corroborating previous findings (14, 15). It is possible that they might never appear independently, and that in cases where only one component of the OLE RNA-OapA partnership is found, the other was missed computationally or the genome assembly was incomplete/inaccurate. In rare instances, we have observed what appears to be defective OLE RNA genes in organisms that lack OapA. Similarly, OLE-containing organisms commonly carry OapB (~60%) and OapC (~45%), but these proteins also appear in some species even when OLE RNA and OapA are absent. Furthermore, OapB and OapC are absent from certain clades of OLE-containing organisms—in particular, the RF39 and TANB77 clades. These results indicate that OapA is indispensable for OLE RNP function, but OapB and OapC are not essential components of the complex in some species. To rationalize their presence in some species, OapB and OapC must have other important functions when OLE RNA is absent.

**Fig 5:**
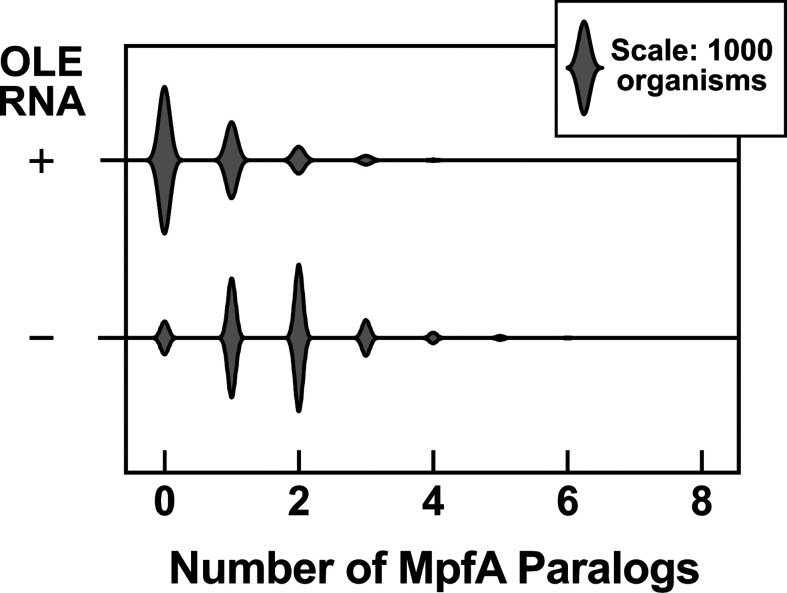
Violin plot depicting the number of MpfA paralogs in species in Bacillota, categorized by the presence or absence of OLE RNA. Plot is derived from the data in Fig. S4.

Although some bacterial species carry both OLE RNA and MpfA, there is a strong tendency for clades to lack MpfA when the organisms have OLE RNP complexes. We counted the number of MpfA paralogs in each genome assembly (Fig. S4) and found that organisms without OLE RNA tend to have several MpfA paralogs (sometimes as many as eight). However, OLE-containing organisms typically have no more than one, if any (Fig. 5; Fig. S4). Again, this observation supports the hypothesis that the OLE RNP complex, or OapA in particular, carries out one or more functions that are similar to those performed by MpfA. Furthermore, perhaps some species employ multiple versions of MpfA to collectively achieve the same spectrum functions as the OLE RNP complex.

### MpfA is structurally and phylogenetically related to OapA

**Fig 6:**
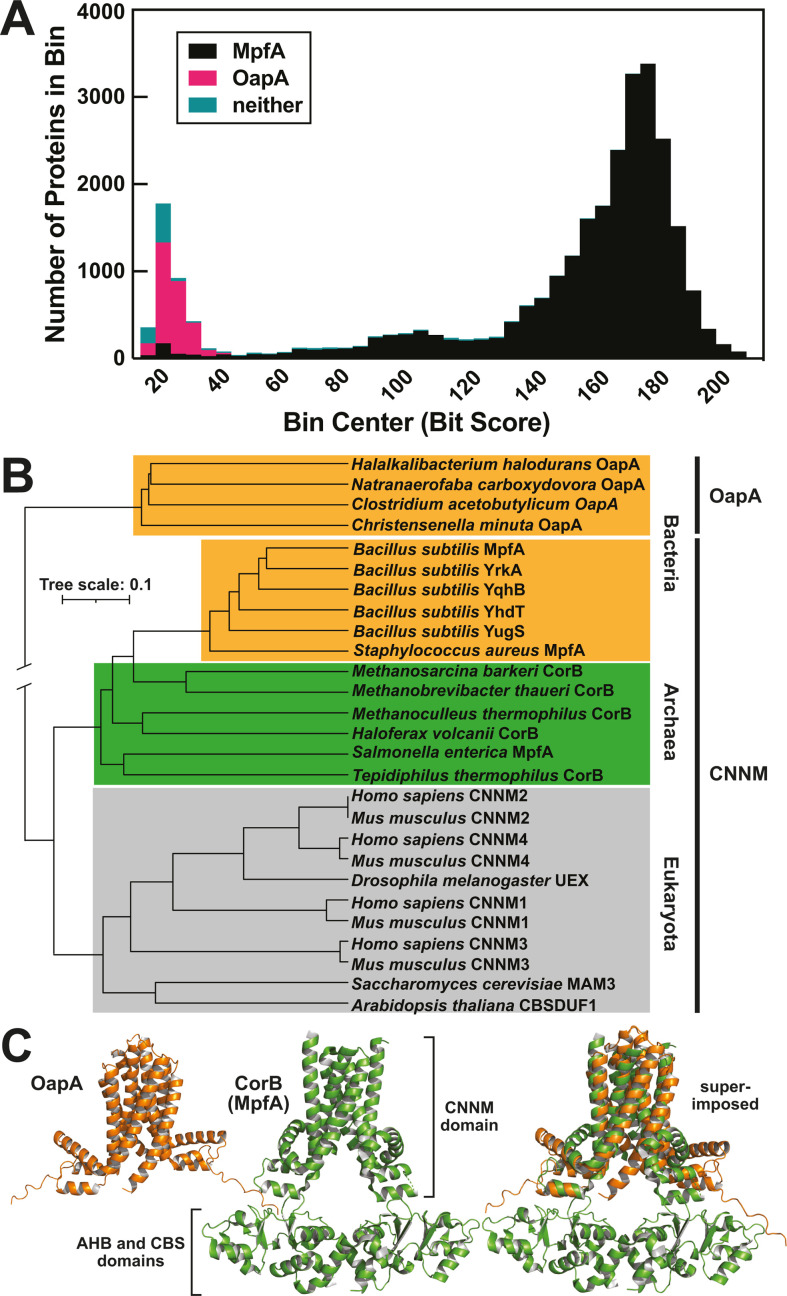
Phylogenetic and structural comparison of OapA and MpfA. (**A**) Histogram of the number of representative proteins with bit scores derived from an HMM search of a database of all proteins from Bacillota using the Pfam HMM of the CNNM domain (PF01595). (**B**) Neighbor-joining phylogenetic tree of OapA proteins and CNNM domains from proteins corresponding to all domains of life. Proteins with names other than OapA and MpfA are close homologs of MpfA. The tree was created using the MAFFT v.7.520 web server (69). Protein sequences were obtained from UniProt (70). The MAFFT alignment was also compared to an alignment performed using Clustal Omega (71). Tree scale bar is given in amino acid substitutions per site. (**C**) Comparison of an AlphaFold2 prediction of *H. halodurans* OapA structure (left), a published crystal structure of *Methanoculleus thermophilus* R235L mutant CorB (PDB: 7M1U) (middle), and an overlay of the two structures (right).

As noted above, we have previously reported similarities between the CNNM domain of MpfA and the sequence of OapA, as well as similarities regarding their biological effects when deleted (18). Proteins with CNNM domains are present in species from all three domains of life, and numerous examples have been experimentally validated to function as metal ion transporters (65, 66), including MpfA (28–30). To further establish the extent of similarity between OapA and the CNNM domain of MpfA, we used the Pfam (68) HMM of the CNNM domain (PF01595) to do a search of all proteins in Bacillota. A histogram of the bit scores from the search reveals that MpfA proteins most closely match the HMM used for the search and that OapA distinctly clusters as the next closest match (Fig. 6A). These protein identity assignments were made using custom HMM models for MpfA and OapA. Proteins assigned as “neither” often form the typical MpfA domain architecture but carry substantial sequence alterations in one or more regions.

Although the CNNM (or CNNM-like) domains of MpfA and OapA are most closely related to each other, there are some key differences. For example, CNNM domains lack a DxxxD motif (Fig. S5), which is a distinctive feature of OapA proteins (15, 18). Furthermore, OapA lacks a highly conserved proline residue that is a characteristic of CNNM domains. A neighbor-joining phylogenetic tree of OapA proteins and CNNM domains from all three major domains of life suggests OapA may be a distant homolog of the CNNM domain that diverged prior to the last universal common ancestor (Fig. 6B).

In addition to these sequence analyses, we also assessed structural similarities between OapA and CNNM-domain-containing proteins. Structures of multiple CNNM-domain-containing proteins exist (65, 66), but there were no published structures of OapA at the time of this analysis. Therefore, we predicted the structure of *H. halodurans* OapA using AlphaFold2 (72). Consistent with the previous conclusion that OLE RNA binds OapA with a 1:2 stoichiometry (15), the structure for OapA appears to be best modeled as a dimer (Fig. 6C). A structural homology search using the Dali server (73) revealed a published crystal structure of an R235L mutant of the *M. thermophilus* MpfA ortholog CorB (PDB: 7M1U) as a top match in the PDB database (74) to the predicted OapA structure. Although each OapA monomer has four transmembrane helices while each CorB monomer has only three, the CNNM domain of the CorB structure overlays well with the predicted OapA structure (Fig. 6C). These results suggest that OapA and MpfA proteins form similar structures, possibly share an evolutionary origin, and perhaps carry out similar functions.

### Concluding remarks

Phylogenetic profiling as applied herein ranks the three known essential protein partners for the *H. halodurans* OLE RNP complex most highly. These data are consistent with the hypothesis that the primal functions of OapA, OapB, and OapC are their participation in forming the OLE RNP complex. However, two of these proteins, OapB and OapC, are not universally required for the function of the complex because some species that carry OLE RNA lack these proteins. Furthermore, if OLE RNA binding were early functions of these three proteins, then some modern species have evolved other biological roles at least for OapB and OapC.

Our analyses also revealed additional proteins that correlate with the presence of OLE RNA (Fig. 4A). These hits thus serve as candidates for a possible “OapD” or a fourth protein that is indispensable for the function of the *H. halodurans* core OLE RNP complex. Currently, none of these candidates have appeared as hits in genetic suppressor selections (17, 18, 23), including those that revealed the other essential components of the complex. Furthermore, we do not observe the additional phylogenetic profiling hits on the list of strong candidates identified by OLE RNA pull-down assays (21). There could be several reasons why phylogenetic profiling candidates do not appear in these other data sets. For example, a protein essential for the function of the OLE RNP complex might also be essential for the survival of the cell under the genetic suppressor selection conditions and thus would be missed. Also, a protein essential for OLE RNP complex function might not (for technical reasons) copurify with OLE RNA when isolating the complex from cells, which is the case for OapA (21).

Although there are no OapD candidates that appear in more than one of the phylogenetic profiling, suppressor selection, and RNA pull-down hit lists, we cannot rule out the possibility that OLE RNA from *H. halodurans* has additional protein partners that are essential for its general function. We do have strong evidence that the OLE RNP complex has more protein partners that are relevant to some of its functions. These additional partners include ribosomal protein S21 (Breaker et al., unpublished). Thus, it seems possible that OLE RNA will form direct interactions with additional proteins, and the proteins identified in this study should be regarded as possible binding partners for OLE RNA, even if their interactions are not essential for the full spectrum of functions performed by the RNP complex.

Even if the OLE-correlated proteins do not directly bind to OLE RNA, they might be associated with pathways and processes relevant to the function of the RNP complex. If true, then the functions of these proteins could provide clues regarding the characteristics of OLE-containing organisms and the ways in which they use OLE RNA. The most broad example of this is the correlation with many proteins involved in sporulation, which suggests that OLE RNA is either involved in sporulation or performs functions that are important for sporulating organisms.

The observation that MpfA strongly anticorrelates with OLE RNA is particularly intriguing, especially considering its sequence and structural similarity to OapA. If the two proteins exhibit some functional equivalency, then it is interesting to consider the possibility that one retains its original function and that it could be considered the evolutionary predecessor of the other. Most large, structured RNAs, such as ribosomal RNAs, RNase P, and self-splicing RNAs, have been proposed to be ancient ribozymes that predate the emergence of proteins in evolution (9–11). Likewise, perhaps OLE RNAs are also of ancient origin and have adapted to interact with certain proteins of modern species to best carry out their diverse biological and biochemical functions. MpfA proteins might then have been derived from OapA and accrued additional protein domains (Fig. 6C) to serve the functions that otherwise would be performed by OLE RNA and its other protein partners. Furthermore, the fact that organisms lacking an OLE RNP complex tend to have more, distinct versions of MpfA proteins (sometimes as many as eight) (Fig. 5; Fig. S4) suggests that more than one type of MpfA protein is needed to replace the function of the RNP complex.

If this proposed evolutionary connection is true, then OLE RNAs might perform functions analogous to those of the tandem CBS domains and HlyC/CorC domain found in MpfA proteins. CBS domains have been implicated in binding ATP and Mg^2+^-ATP (65, 66), whereas the precise function of HlyC/CorC domains remains to be established. A more detailed mechanistic understanding of the function and properties of MpfA and its CBS and HlyC/CorC domains could help in the effort to define the biochemical functions of OLE RNA and vice versa. Likewise, the ubiquity and high level of redundancy of MpfA proteins suggest that both MpfA and OLE RNA are important for managing key processes relevant to stress mitigation.

## MATERIALS AND METHODS

### Structured RNA searches

Infernal 1.1.4 (36) was used to search a database of all genomes in GTDB R08-RS214 (34) using a previously published Stockholm file for OLE RNA as a query (21). Separate histograms were plotted for hits deemed by Infernal to be truncated or nontruncated. A histogram of nontruncated hits was plotted of bit scores of all hits. The histogram was used to set cutoffs for the categories of RNAs. Nontruncated hits with a bit score of 390.0 or higher were designated “full-length” OLE RNA hits. Nontruncated hits with a bit score of at least 310.0 and less than 390.0 were designated “variant 1.” Nontruncated hits with a bit score of at least 235.0 and less than 310.0 as well as truncated hits with a bit score of at least 235.0 were designated “variant 2.” The Stockholm files were used to build consensus sequence and secondary structure models using R2R 1.0.6 (75) for full-length, variant 1, and variant 2 OLE RNAs (Fig. 1).

### Phylogenetic species tree construction

Genome assemblies containing OLE RNA were mapped onto the GTDB R08-RS214 tree using the R package ape v5.7-1 (76). OLE RNA sequences were detected in 2,846 assemblies. Of these, 2,822 were in the Bacillota phylum, but 24 of them were from distantly related taxa. These latter hits were discarded because we suspected them to have arisen from assembly or taxonomy assignment errors, as explained in the main text. Utilities from the R packages ape v5.7-1 and TreeTools v1.10.0 (77) were used to prune the tree to generate the smallest subtree containing all OLE-containing organisms. The tree was visualized using iTOL v6 (78).

### Protein database construction

The NCBI FTP (42) was used to download all protein sequences from all genome assemblies in the OLE-centric bacterial species tree. For assemblies without protein sequence FASTA files from the NCBI FTP, Prodigal v2.6.3 (43) was used in “normal mode” to predict protein sequences.

### Protein homology searches

All protein sequences from *H. halodurans* and *B. miscanthi* were used as queries to perform DIAMOND v2.1.7.161 (44) searches against the full protein database with parameters “--evalue 1e-10” and “--very-sensitive.” The *e*-value cutoff of 1 × 10^−10^ was chosen empirically because it robustly separates most likely homologs without accepting too many non-homologous proteins across a variety of query proteins. For each protein from the query, the set of all DIAMOND search hits was taken as a protein family. For each protein family, the proportion of OLE-containing and OLE-lacking organisms, respectively, that contain at least one member of the protein family was calculated using a custom Python script.

### Protein clustering

Proteins were clustered using MMseqs2 Release 14 (79) using a cascaded clustering strategy inspired by a strategy used in a previous publication (80). Four clustering steps were performed with a representative from each step carried on to the next clustering steps. The first clustering step was performed with default parameters. The second through fourth steps used the “-s 7.5” and “-c 0.7” flags. The second step additionally used the “--cov-mode 5” flag and the fourth step additionally used the “--cluster-mode 1” flag. Final clusters were calculated from all the steps, and clusters with fewer than five proteins were discarded.

### Mutual information calculation

Mutual information for each protein family was calculated using the standard formula for mutual information (81, 82). Specifically, mutual information is given by $I\left(X;Y\right)=\sum_{x,y}p\left(x,y\right)\log\frac{p\left(x,y\right)}{p\left(x\right)p\left(y\right)}$ for random variables *X* and *Y* distributed jointly by p(x,y). We used log base 2 as a convention. Let *X* and *Y* be random variables that take the values 1 or 0 if a genome assembly does or does not have *ole* or a gene for the protein in question, respectively, and $p_{x,y}$ be the probability that *X* and *Y* take values of x and y, respectively. By the aforementioned formula, the mutual information is given by $p_{0,0}\log_{2}\frac{p_{0,0}}{\left(p_{0,0}+p_{0,1}\right)\left(p_{0,0}+p_{1,0}\right)}+p_{0,1}\log_{2}\frac{p_{0,1}}{\left(p_{0,0}+p_{0,1}\right)\left(p_{0,1}+p_{1,1}\right)}+p_{1,0}\log_{2}\frac{p_{1,0}}{\left(p_{0,0}+p_{1,0}\right)\left(p_{1,0}+p_{1,1}\right)}+p_{1,1}\log_{2}\frac{p_{1,1}}{\left(p_{1,0}+p_{1,1}\right)\left(p_{0,1}+p_{1,1}\right)}$. Calculations were discarded in any case for which the operand of the logarithm was undefined or zero.

### HMM construction and searches

Protein sequences of OapA (WP_010898928.1), OapB (WP_053432310.1), OapC (WP_010896311.1), and MpfA (NP_388836.1) were used as queries to perform sequence homology searches against the aforementioned protein database using BLAST+ 2.13.0 (83) with the flag “-max_target_seqs 200000” and default values for all other parameters. Strict bit score cutoffs were chosen for each search to select the most likely homologs. These cutoffs were chosen using a combination of analyzing histograms of bit scores and the gene context of selected hits. For OapC, special care, including stricter cutoffs and a more thorough analysis of selected hits, was taken to ensure the selection of homologs of YbxF (OapC) and not its paralog YlxQ, which cannot substitute for OapC in *H. halodurans* (17, 21). For each protein family, hits scoring above the respective selected bit score threshold were aligned using MAFFT v7.505 (84) with the FFT-NS-2 alignment method in all cases. HMMER 3.3.2 (85) was then used to create HMMs from each alignment using hmmbuild with default parameters. HMM searches were performed using hmmsearch with default parameters. Histograms of bit scores were calculated and plotted in GraphPad Prism 10.

### Protein structure prediction and alignment

The *H. halodurans* C-125 OapA protein sequence (WP_010898928.1) was run on the AlphaFold Colab notebook (72). The resulting structure was manually modeled as a dimer using PyMOL (The PyMOL Molecular Graphics System, Version 2.0 Schrödinger, LLC). Structural homology search against PDB (74) was performed using Dali (73), which reported an RMSD of 6.2 and 2.9 Å for the A and B chains, respectively, of the *M. thermophilus* R235L mutant CorB structure (PDB: 7M1U). The OapA and CorB structures were aligned using the “super” command in PyMOL. All protein structure images were created using PyMOL.

## SUPPLEMENTAL MATERIAL

Supplemental File msphere.00159-24-s0001.docx Supplemental text and figures.

ASM does not own the copyrights to Supplemental Material that may be linked to, or accessed through, an article. The authors have granted ASM a non-exclusive, world-wide license to publish the Supplemental Material files. Please contact the corresponding author directly for reuse.
