# Supplementary material for: Bioinformatic prediction of proteins relevant to functions of the bacterial OLE ribonucleoprotein complex
Source: mSphere. 2024 May 21;9(6):e00159-24. doi: 10.1128/msphere.00159-24 (PMC11332333; doi:10.1128/msphere.00159-24)
Supplement: Supplemental File — Supplemental text and figures. [file msphere.00159-24-s0001.docx]

**Supplemental Information**

**Bioinformatic prediction of proteins relevant to functions of the bacterial OLE ribonucleoprotein complex**

Chrishan M. Fernando^1^ and Ronald R. Breaker^1,2,3,*^

^1^Department of Molecular Biophysics and Biochemistry, Yale University, New Haven, CT 06511-8103, USA

^2^Department of Molecular, Cellular and Developmental Biology, Yale University, New Haven, CT 06511-8103, USA

^3^Howard Hughes Medical Institute, Yale University, New Haven, CT 06511-8103, USA

^*^Address correspondence to Ronald R. Breaker, [ronald.breaker@yale.edu](mailto:ronald.breaker@yale.edu)

**Phylogenetic Profiling Details**

The phylogenetic profiling strategy used in this study has two major advantages over previous protocols. First, because GTDB is used as a representative database of the known bacterial tree of life, the resulting phylogenetic profiles are more representative of protein abundance in nature. Furthermore, the analysis was performed on 17,916 species, which far exceeds the number of species used in most previous analyses. This is primarily made possible by the use of the ultra-fast sequence homology search algorithm DIAMOND (1) to generate protein families. We also tried other tools like MMseqs2 (2). However, we found that clustering algorithms like MMseqs2 were too conservative to generate protein families reliable enough for phylogenetic profiling by themselves. For instance, we had difficulty clustering most of the likely OapB sequences together using MMseqs2. Therefore, for the ‘reference agnostic’ phylogenetic profiling strategy, MMseqs2 was used as an initial step to reduce the number of redundant proteins from the same family but then DIAMOND was used to generate final protein clusters. Additionally, it was difficult to appropriately tune the MMseqs2 parameters without either under-clustering and overrepresenting certain proteins families or over-clustering and completely missing entire protein families that were incorporated into clusters with other protein families.

HMM searches were also tried because such methods are more sensitive than sequence homology-based methods like DIAMOND and MMseqs2 (3). However, HMM searches at the scale required for this study were far slower than DIAMOND. Furthermore, such searches rely on curated databases of HMMs, but many potential protein families of interest might not have publicly available HMMs. For instance, HMMs for the OLE-associated proteins OapA, OapB and OapC did not exist prior to this project. Regardless, the use of more accurate and sensitive methods for generating protein families could potentially uncover correlations that otherwise would not be apparent.

Ultimately, our results demonstrate that phylogenetic profiling is a feasible method for predicting protein partners of large ncRNAs. Beyond investigating OLE RNA, this approach could prove useful in identifying protein partners of many other poorly characterized bacterial ncRNAs.

**References**

1. Buchfink B, Reuter K, Drost H-G. 2021. Sensitive protein alignments at tree-of-life scale using DIAMOND. *Nat Methods* 18:366-368.
2. Steinegger M, Söding J. 2017. MMseqs2 enables sensitive protein sequence searching for the analysis of massive data sets. *Nat Biotechnol* 35:1026-1028.
3. Eddy SR. 2011. Accelerated Profile HMM Searches. *PLOS Comput Biol* 7:e1002195.


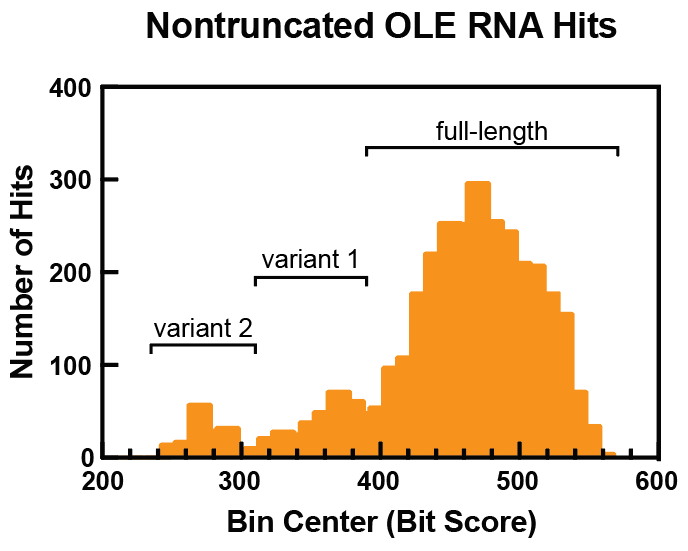


**Figure S1.** Histogram of bit scores from the Infernal search for OLE RNA sequences in all GTDB R08-RS214 genomes. Regions of the graph are delimited based on thresholds described in the Materials and Methods section that designate OLE RNA hits as ‘full-length’, ‘variant 1’, or ‘variant 2’.


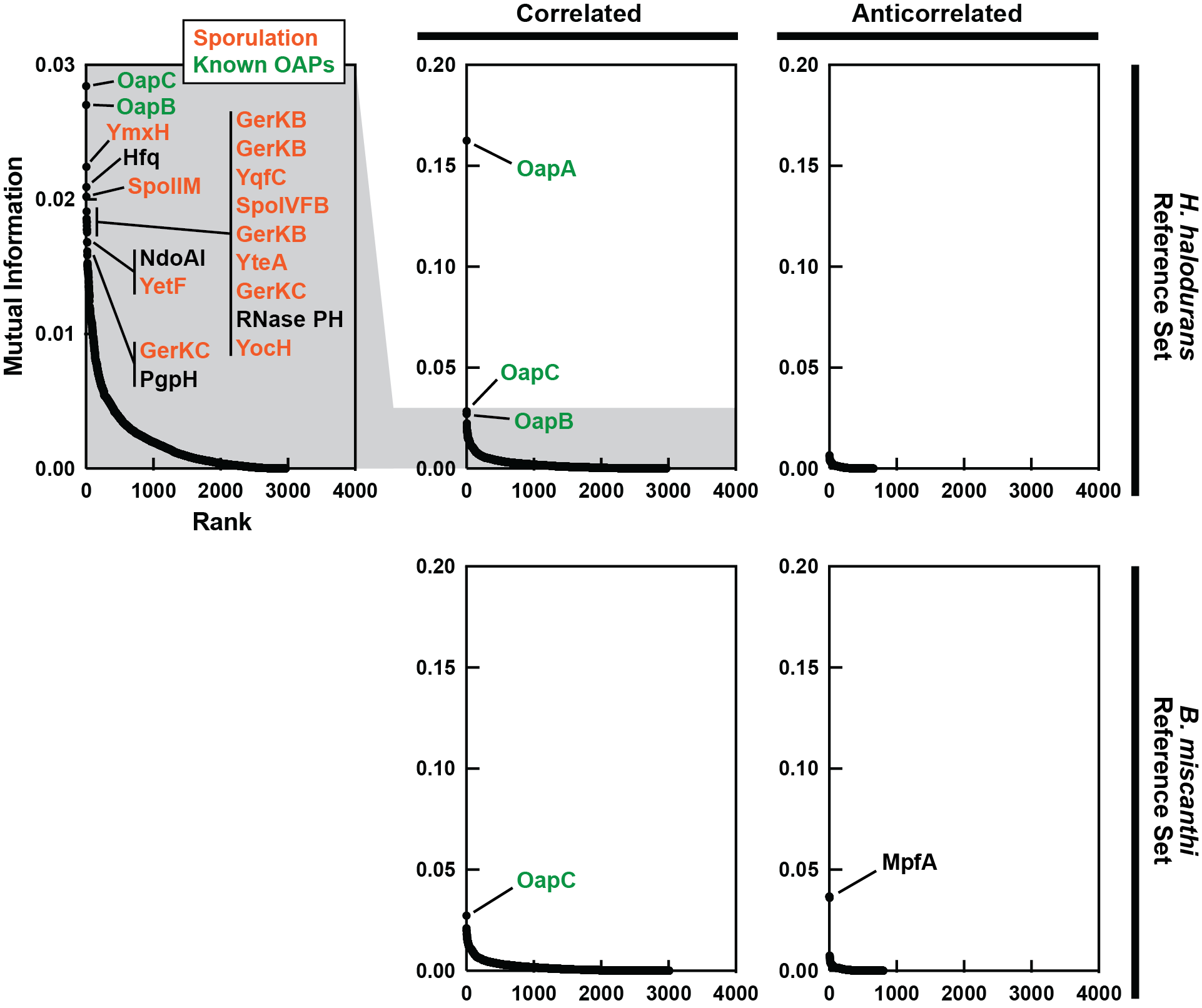


**Figure S2.** Plots of mutual information calculations between OLE RNA presence and the presence or absence of a given protein among all analyzed genome assemblies. Calculations were performed as described in Materials and Methods.


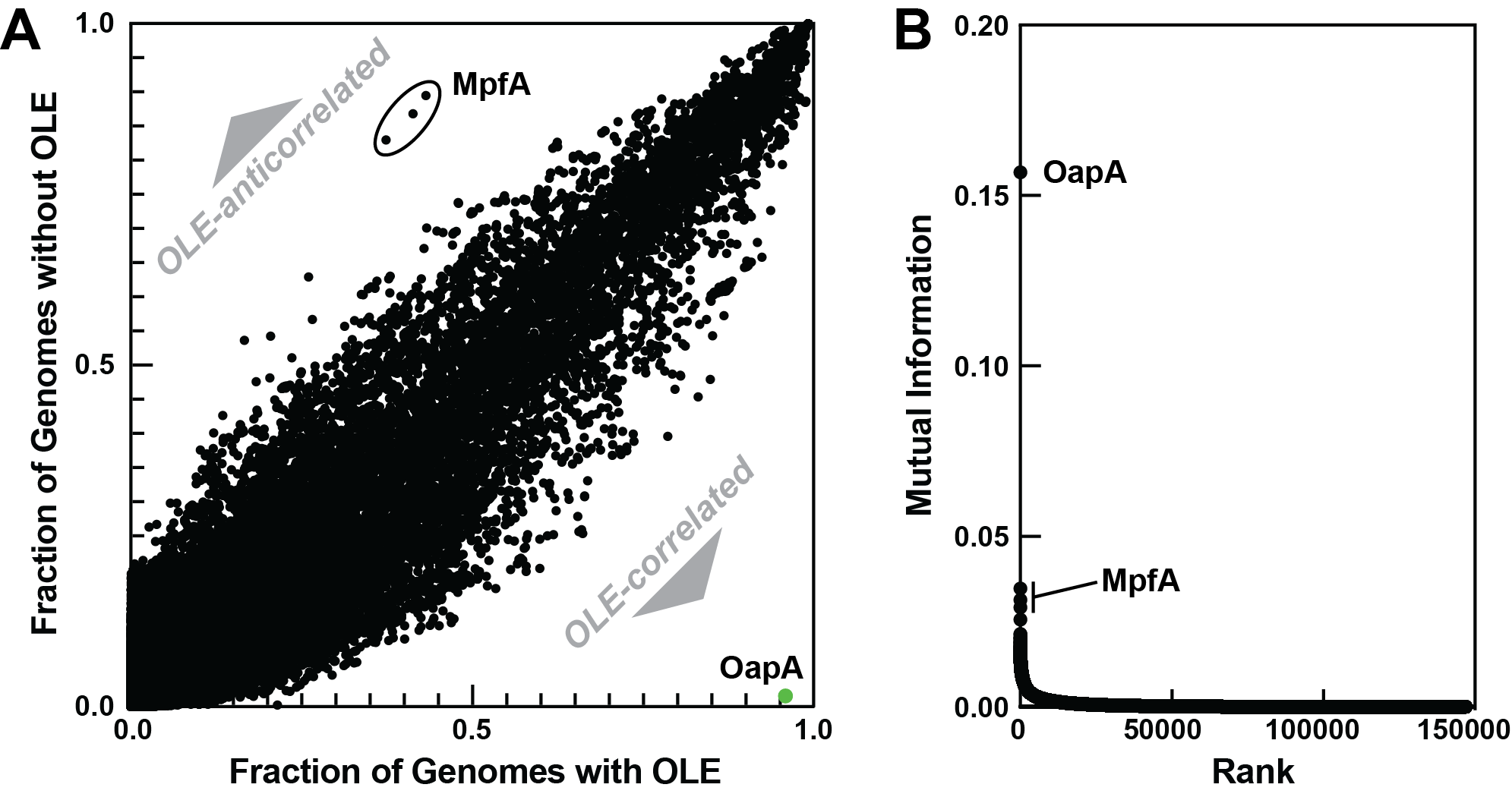


**Figure S3.** ‘Reference agnostic’ phylogenetic profiling analysis of protein correlation with OLE RNA. (**A**) Plot of proteins correlated and anticorrelated with OLE RNA as determined by the reference agnostic phylogenetic profiling analysis strategy. Annotations are as described for **Fig. 4**. Note that OapB and OapC do not appear on the plot because the clustering process used for this analysis grouped them with other similar proteins. (**B**) Plot of mutual information calculations for protein candidates identified in A.


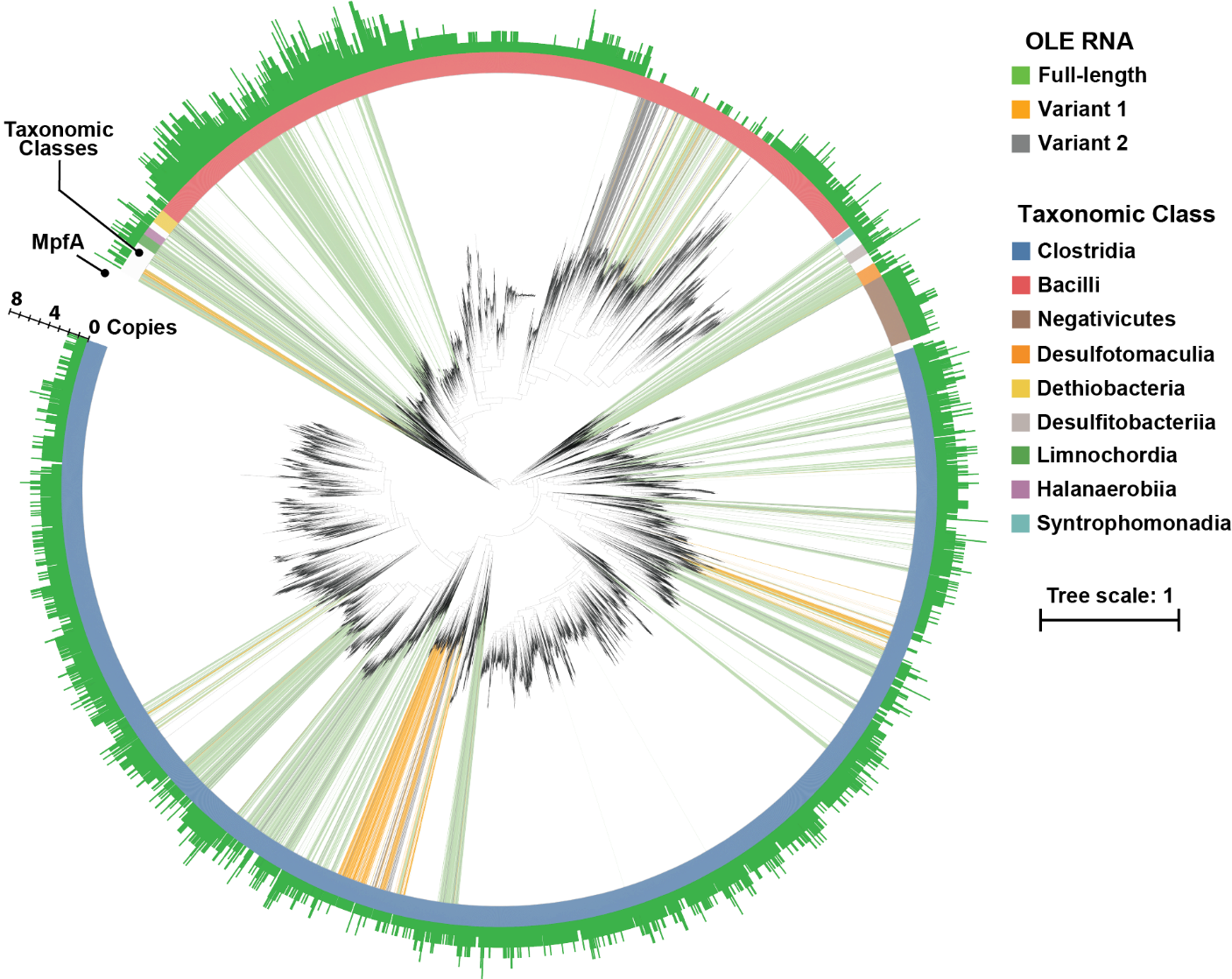


**Figure S4.** Phylogenetic species tree of Bacillota derived from GTDB R08-RS214 depicting the distributions of OLE RNA and MpfA. The tree, scale bar, and annotations are as described in **Fig. 2**.


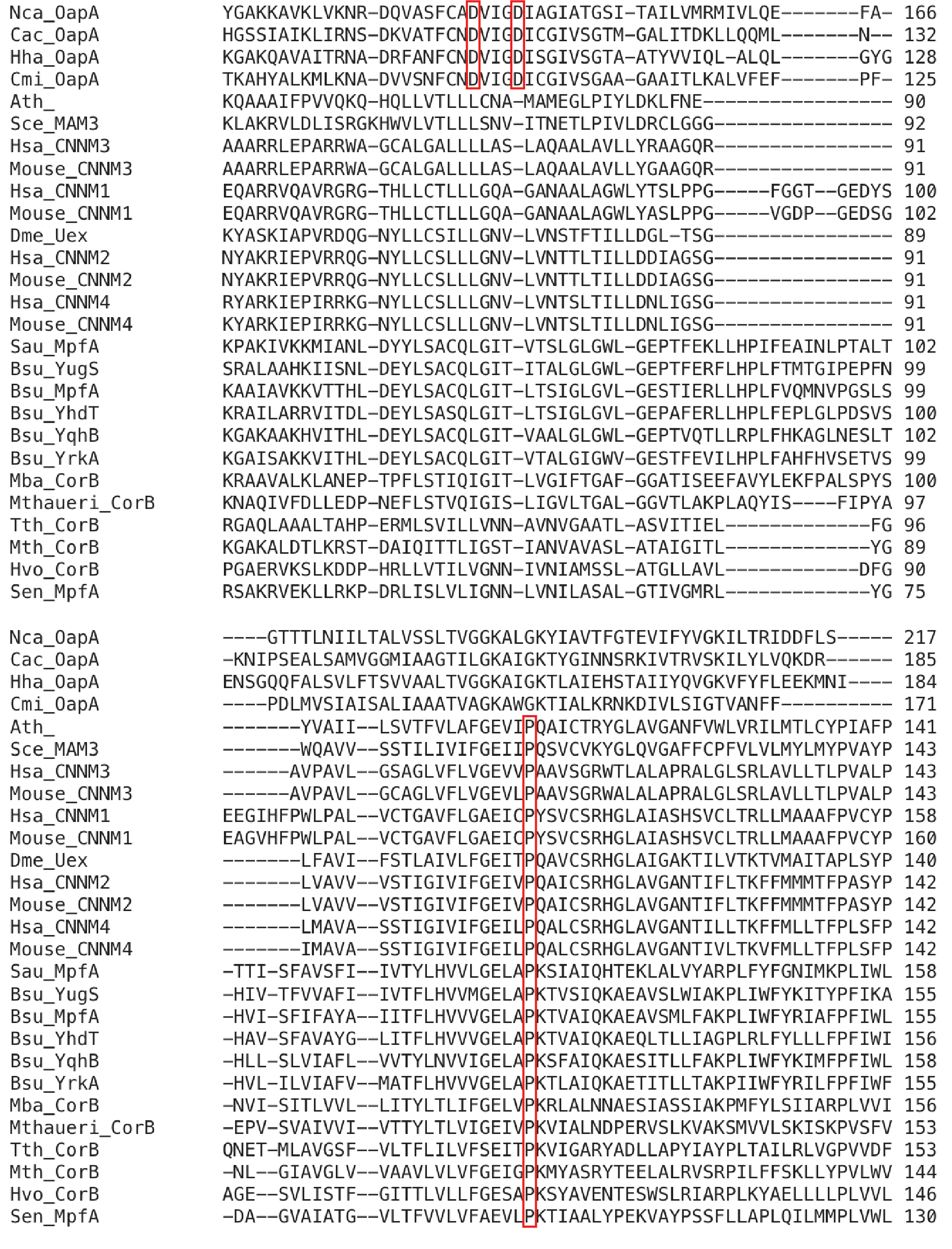


**Figure S5.** Multiple sequence alignment of OapA proteins and the CNNM domains of various proteins from across all domains of life. Sequences were obtained from UniProt (84) and aligned using the EMBL-EBI Clustal Omega (85) web server. Red boxes designate conserved amino acids discussed in the main text.
